# Progress of the Gulf Cooperation Council (GCC) Countries Towards Achieving the 95-95-95 UNAIDS Targets: A Review

**DOI:** 10.1007/s44197-023-00097-1

**Published:** 2023-04-20

**Authors:** Salah Al Awaidy, Ramy Ghazy, Ozayr Mahomed

**Affiliations:** 1 Office of Health Affairs, Ministry of Health, Muscat, Oman; 2 Tropical Health Department, High Institute of Public Health, Alexandria University, Alexandria, Egypt; 3 Department of Public Health Medicine, University of KwaZulu Natal, Durban, Howard College Campus South Africa; 4 Dasman Diabetes Institute, Kuwait City, Kuwait

**Keywords:** UNAIDS 95-95-95 targets, HIV, Antiretroviral therapy, Viral suppression, GCC countries

## Abstract

**Background:**

In 2014, the Joint United Nations Programme on HIV/AIDS (UNAIDS) and partners launched the 90-90-90 targets. These were further updated to correspond to 95-95-95 by the year 2025. We present an overview of the progress made by Gulf Cooperated Council (GCC) countries towards meeting the global targets.

**Methods:**

We extracted data from Global AIDS Monitoring (GAM), UNAIDS AIDS Info, HIV case reporting database, and the WHO global policy uptake for six countries: Bahrain, Kuwait, Oman, Qatar, Saudi Arabia and the United Arab of Emirates (UAE) to assess the HIV/AIDS burden in the six GCC countries, and the progress towards achieving the 95-95-95 goal.

**Results:**

By the end of 2021, an estimated 42,015 people living with HIV (PLHIV) were residing in the GCC countries with prevalence levels below 0.01%. Data from four GCC countries, Bahrain, Oman, Qatar and UAE, indicated that by 2021, 94%, 80%, 66%, and 85% of HIV-positive population knew their status, respectively. 68%, 93% (2020 data), 65%, 58% and 85% of PLHIV in Bahrain, Kuwait, Oman, Qatar and UAE who knew their status were on anti-retroviral therapy (ART), respectively, and 55%, 92%, 58% and 90% (2020 data) among those who were on ART had viral suppression in Bahrain, Kuwait, Oman and KSA, respectively.

**Conclusion:**

The GCC countries have made great strides toward fulfilling the 95-95-95 targets, but the interim 2025 overall UNAIDS targets remain unmet. The GCC countries must strive diligently to accomplish the targets by emphasising early identification of the cases by enhanced screening and testing, as well as prompt commencement of ART therapy with viral load suppression.

## Introduction

In 2019, World Health Organization (WHO) and Joint United Nations Programme on HIV/AIDS (UNAIDS) estimated that 38.4 million people worldwide were living with HIV/AIDS (PLHIV) and in 2021, 650,000 people died as a result of HIV/AIDS-related causes [1]. Furthermore, in 2019 an estimated 1.5 million people were infected by HIV [2].

Epidemiologic investigations of the global burden of HIV/AIDS have shown the disproportionate toll of the disease in various regions of the globe, the WHO African region contributing to nearly 60% of the new global HIV infections in 2020 [2]. Additionally, some regions including the Eastern Europe, Central Asia, Western and Central Africa and the Middle East North Africa (MENA) region are still trailing in terms of controlling HIV infections and the scarcity of reports investigating HIV/AIDS [3–5], demonstrates difficulties in ART connection and retention.

In 2021, globally 85% [75–97%] of people living with HIV recognized their status, 75% [66–85%] were receiving treatment, and 68% [60–78%] were virally suppressed [6].

In the Eastern Mediterranean Region (EMR), the HIV/AIDS epidemic has been increasing. In 2020 the EMR witnessed a 24% increase in new HIV infections and 12% increase in AIDS related deaths compared to 2015. In 2020, WHO and UNAIDS estimated that 420,000 people were living with HIV (PLHIV) including 14 000 children. Out of them 42% were diagnosed (1st 90); 27% were receiving treatment out of those who were diagnosed (2nd 90), and out of PLHIV who were receiving treatment, 27% achieved viral load suppression [7].

Numerous studies have indicated that early access to antiretroviral therapy (ART) decreases viral load to extremely low or undetectable levels, leading to improved health outcomes among PLHIV [8–11]. In addition, this strategy can help to minimize the risk of forward transmission of the virus; therefore, this approach serves as the basis for the Universal Test and Treat (UTT) guidelines [12, 13]. Based on these recommendations, the number of PLHIV on anti-retroviral therapy (ART) has increased globally, with 28.2 million people accessing ART, as of June 2021 [14]. However, approximately 73% of all PLHIV receive ART, with disparities in access to ART treatment based on age, gender, and geographic location [14]. In 2020, 74% (7–90%) of adults aged 15 years and older and 54% (37–69%) of children younger than 14 years of age with HIV infection had access to treatment [14]. Globally, females had higher access to ART treatment with 79% (61–95%) adult females having access as against 68% (52–83%) of adult males [14]. Disparities in access to ART is also observed across different countries worldwide [15].

UNAIDS has proposed new ambitious proposed global targets of 95-95-95 by 2025, which would make a substantial impact for those with HIV. In this strategy, 95% of all people living with HIV will know their HIV status, 95% of all people with HIV infection will receive sustained ART and 95% of all people receiving ART will have viral suppression (< 1000 copies/mL) [16]. These targets have been endorsed and adopted by all countries with an accountability towards achievement by the year 2025.

Measuring progress toward the 95-95-95 targets set by UNAIDS is critical for measuring success toward reversing the HIV epidemic and is viewed as the cornerstone for efforts to halt the HIV/AIDS epidemic [17].

COVID-19 pandemic posed new challenges to the 90-90-90 targets; widespread lockdowns and restrictions disrupted HIV testing and diagnosis and hindered access to ART that resulted in non-adherence to treatment [18]. In 2020, of all PLHIV, 84% (67–> 98%) knew their positive status, 73% (56–88%) were accessing ART and 66% (53–79%) were virally suppressed [19].

The Gulf Cooperation Council (GCC) countries (Bahrain, Saudi Arabia (SA), Kuwait, Oman, Qatar, and the United Arab of Emirates (UAE) are high-income countries, are part of Eastern Mediterranean Region (EMR), with an overall HIV prevalence rate of 0.1% among adults aged 15 to 49 years, one of the lowest prevalence rates worldwide [20].

In 1987, the GCC countries National AIDS Programs (NAP) was founded, and an HIV/AIDS unit or department was established as part of the Ministries of Health in the countries of GCC. The diagnosed individuals are referred to HIV treatment clinics where they would be treated and cared for the rest of their lives. The program provides ART free of charge to all patients in all tertiary and secondary hospitals in the provinces.

However, there is lack of published research describing the progress of the six GCC countries towards achieving the global 95-95-95 targets. We herewith aim to provide an overview on the progress of GCC countries towards achieving the 95-95-95 targets using data obtained from the member states. Correspondingly, the GCC countries could revise their national action plan to achieve the 95-95-95 HIV-targets and march towards its elimination by 2025.

## Methodology

### Study Design and Data Collection

We conducted a secondary data analysis of data collected between 2015 and 2021 in six GCC countries namely Bahrain, Kuwait, Qatar, Oman, Saudi Arabia (SA) and the United Arab of Emirates (UAE). Data sources included; (1) HIV/AIDS epidemiological estimates published by UNAIDS between 2015 and the end of 2021; (2) HIV case reporting data submitted to WHO EMR as part of HIV case reporting data; and (3) HIV policy analysis published by WHO [21].

For coverage indicators of 95-95-95 targets, we compared the data from 2015 with those of 2021 to report on the progress made in each area [20]. We also collected data on the proportion of people living with HIV will know their HIV status, proportion of all people with HIV infection to receive sustained ART and proportion of all people receiving ART will have viral loads suppression.

### Data Extraction and Analysis

We categorized the input from these data sources to inform the following: (1) burden defined as HIV prevalence, estimated PLHIV, new HIV infections and AIDS related deaths; (2) progress towards the global targets defined as (a) proportion of PLHIV who know their HIV status out of all PLHIV, (b) proportion of PLHIV who are receiving treatment out of those who know their HIV status; and (c) proportion of those who achieved viral load suppression out of those who are receiving treatment; (3) policy of the GCC countries on testing, treatment and virus suppression. For testing, indicators included availability of a policy on testing provision free to all, if partner notification included in the testing policy, and if the country has a policy on HIV self-testing. For treatment, indicators included if the country adopted treat all policy, rapid initiation of ART and the implementation status of recommended ART initiation. For viral load suppression, indicators included availability of national policy on routine viral load testing, and its implementation status.

We compared 2015 with 2021 data to report on the progress made in each area.

### Statistical Analysis

The patterns of each selected indicator and their change were represented numerically and graphically with bar chart/line plotted using line graph points on the X–Y axis, where X is the time in year (mostly from 2015 to 2021) and Y is value of the selected indicator for each year in number or proportion. The figures were built using Microsoft Office 2016 and ggplot2 package built under R version 4.2.

### Ethics Approval

We used anonymized secondary data and thus, ethical study approval was not required as this study was based on secondary data extracted from national data, from official domains and adheres to the guidelines of the Helsinki Declaration.

## Results

### Burden of HIV/AIDS in the GCC Countries

#### Incidence of HIV Infection

Between 1990 and end of 2021, the incidence of HIV increased steadily in GCC countries from 0.39 to 0.52%, after which it decreased to 0.19% in 2020. On the other hand, the MENA region exhibited a significant increase from below 0.01% in 1990 to 0.06% in 2001, which remained constant until 2006, then the incidence steadily decreased to 0.03% in 2012, and remaining stagnant until 2020. Interestingly, over the years until 2021 the general HIV incidence in Bahrain, Kuwait and Oman remained < 0.1%. Between years 1993 and 2020, Saudi Arabia witnessed a gradual increase in HIV incidence from < 0.01 to 0.05%. In contrast, the incidence in Qatar followed a different pattern: it was 0.02% in 1990, then dropped to 0.01% in 1992, and stayed constant until 1999, at which point it began to steadily rise to 0.04% in 2004 before increasing significantly in 2019–2020 to reach 0.06% and 0.07%, respectively. Worth noting that the incidence of HIV in the UAE has increased over the last ten years and reached 0.13% in 2020 from 0.04% in 2011**.**

### HIV Prevalence

Since 1990, the worldwide prevalence of HIV has more than doubled from 0.3% to 0.7%. The HIV prevalence rate in the MENA region, however, has remained constant at less than 0.1% and similar to the MENA region, all GCC countries recorded prevalence levels below 0.1% between 1990 and 2021 (Table 1).

**Table 1:** GCC countries HIV burden, 2015 and 2021

	Prevalence of HIV among adult aged 15 to 49 (%)		Estimated number of people (all ages) leave with HIV (PLHIV)		Estimated number of adult and children newly infected with HIV		Newly reported HIV cases		Estimated number of people dying from HIV-related cases	
	2015	2021	2015	2021	2015	2021	2015	2021	2015	2021
Bahrain	< 0.1	0.1	237	< 500	< 100 [< 100–< 100]	< 100 [< 100–< 100]	20^a^	37	< 100 [< 100–< 100]	< 100 [< 100–< 100]
Kuwait	0.1 [< 0.1–< 0.1]	0.1 [< 0.1–< 0.1]	346	66	NA	NA	35	66	2	NA
Oman	0.08^c^	0.2^d^	2000 [1800–2100]	2500 [2300–2700]	135^b^	147^b^	142	202	< 100 [< 100–< 100]	< 100 [< 100–< 100]
Qatar	0.1 [0.1–0.1]	0.2 [0.1–0.2]	< 200 [< 200–< 500]	< 500 [< 500–< 500]	< 100 [< 100–< 100]	< 100 [< 100–< 100]	< 100 [< 100–< 100]	< 100 [< 100–< 100]	< 100 [< 100–< 100]	< 100 [< 100–< 100]
Saudi Arabia (SA)	< 0.1 [< 0.1–< 0.1]	< 0.1 [< 0.1–< 0.1]	7500 [6700–8200]	12 000^d^ [10 000–14 000]	< 750 [640–920]	1000 [750–1503	599 [510–730]	800 [590–1203]	< 100 [< 100–< 100]	< 200 [< 100–< 200]
United Arab of Emirates (UAE)	0.08	0.1	< 500 [< 500–< 500]	540 [< 500–640]	< 100 [< 100–< 100]	< 100 [< 100–< 100]	49^a^	< 100 [< 100–< 100]	< 100 [< 100–< 100]	< 100 [< 100–< 100]

*NA* not available

^a^Data 2016

^b^Oman Medical Journal [2019], Vol. 34, No. 1: 1–8

^c^Annual Statistical Report, 2021

^d^Data 2020

### HIV in Key Populations at Higher Risk

In the GCC countries, concentrated epidemics with sustained transmission at high HIV prevalence were observed among Injection drug users (IDUs), men who have sex with men, and female sex workers. Unsafe injections remain common in the countries and continue to contribute to new HIV cases. By 2022, the published data indicated a concentrated epidemic in GCC countries among intravenous drug users (IDUs). The prevalence of HIV among IDUs was 4.6%, 0.8%, 11.8%, and 9.8% in Bahrain, Kuwait, Oman and SA respectively [22]. We were unable to disaggregate the data by sub-population as this data is not available. Furthermore, the data has not been disaggregated by population group.

### PLHIV in the GCC Countries

By the end of 2021, it was estimated that 42,015 PLHIV were living in the GCC countries. Of the total number of PLHIV, SA had the highest percentage (27,100; 65%) followed by Oman (9,985; 24%) and the UAE (2,540; 6%). Data extracted for the three countries of Kuwait, Oman and SA showed that males between 25 and 49 years of age accounted for the highest percentage of PLHIV (47%), followed by females between 25 and 49 years of age (22%) and followed by males above the age of 50 years (20%).

Bahrain recorded 237, 548 and 244 cases in 2017, 2018 and 2019 respectively. Kuwait reported 544 cases in 2017 (data for one year only). Comparatively, the number of PLHIV in Qatar rose from fewer than 100 in 2008 to less than 200 in 2018 but remained unchanged until 2020 (200 cases). Oman recorded 2780 cases in 2016, 2920 in 2017, with a progressive decline to 1826 in 2020. In SA, the documented number of PLHIV has continued to grow from fewer than 500 in 1992 to 12,000 in 2020. From 1990 to 2008 there were less than 100 PLHIV cases in the UAE then from 2009 to 2012, this rose to 200. In 2013 documented cases increased to nearly 500 where they remained for six years then climbed to 540 in 2020.

### AIDS-Related Deaths

Globally, between 1990 and 2005, HIV-related deaths grew steadily, rising from 32,000 in 1990 to 1.9 million by 2005, before dropping precipitously to 680,000 by 2021.

**Fig. 1:**
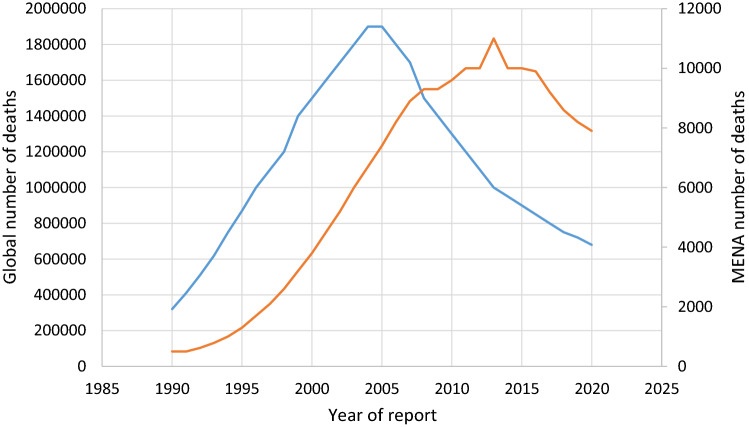
AIDs related deaths: 1990–2025 globally and the in middle East North Africa Region (MENA)

The MENA region's mortality toll peaked in 2013, eight years after the worldwide trend peaked. There were 11,000 recorded fatalities in 2013, however the trend indicated a slow reduction, down to 7,900 in 2020 (Fig. 1), except for SA, where the death toll exceeded 200 in 2021. The death toll in the three GCC countries of Qatar, Oman, SA and UAE, and was below 100 in 2021. There are no data on HIV mortality in Kuwait.

### Progress Towards the 95-95-95 in the GCC Countries

#### First 95: Proportion of Patients Diagnosed with HIV Knowing Their HIV Status

Between 2015 and 2021, Table 2 shows the number of people who are aware of their HIV status in four of the six GCC countries. In Bahrain, of the estimated HIV-infected population, the number of people who were aware of their status of HIV rose from 41% in 2015 to 94% in 2021. Correspondingly, the proportion in Oman fell from 87 to 80% whereas in Qatar, the proportion rose from 41 to 66%. In UAE, the proportion rose from 84 to 85%.

**Table 2:** HIV cascade of care in the GCC countries

	Percent of people living with HIV who know their Status		Coverage of people living with HIV receiving ART		Percent of people living with HIV who have suppressed viral loads	
	2015	2021	2015	2021	2015	2021
Bahrain	41 [36–44]	94 [81–> 98]	33 [29–36]	68 [59–79]	… […–…]	55 [48–63]
Kuwait	… […–…]	… […–…]	73 [57–69]	93^a^	84 [68–75]	92^a^
Oman	87 [80–94]	80 [72–87]	47 [43–51]	65 [58–71]	35 [32–38]	58 [52–64]
Qatar	41 [32–49]	66 [52–78]	41 [32–49]	58 [46–69]	… […–…]	… […–…]
Saudi Arabia	… […–…]	.. […–…]	… […–…]	.. […–…]	85	90^a^
United Arab Emirates	84 [69–78]	85^a^	87^a^	85^a^	97 [89–93]	… […–…]

^a^2020 data

#### Second 95: Proportion of Patients on ART (Out of PLHIV Aware of Their Status)

By 2021, 68%, 93%, 65%, 58% and 85% of PLHIV in Bahrain, Kuwait, Oman, Qatar and UAE were on ART, respectively (Table 2).

#### Third 95: Proportion of Patients on ART that are Virally Suppressed

In 2021 Bahrain, Kuwait, Oman, and SA, 55%, 92%, 58% and 90% of patients, respectively, on ART were virally suppressed (Table 2).

### HIV/AIDS Policies for GCC Countries (Table 3)

**Table 3:** Summarized the HIV testing, treatment and viral suppression for the GCC countries

	Testing			Treatment			Viral suppression		
	Policy on HIV testing provision free to all	Assisted HIV partner notification included in national policy	Availability of a national policy on HIV self-testing	Policy promoting community ART delivery	Country adopted WHO 2017 recommendation on rapid initiation on ART	Implementation status of recommended ART CD4 initiation threshold	National policy on routine viral load testing for adults and adolescents	Implementation status of policy on routine viral load testing	Percentage of ART facilities with viral load testing
Bahrain	Yes	No	No	No	Yes, rapid initiation within 7 days of HIV diagnosis	Implemented countrywide (> 95% of treatment sites)	Yes	Fully implemented	100%
Saudi Arabia (SA)	Yes	No	No	No data available	Yes, rapid initiation within 7 days of HIV diagnosis	Implemented countrywide (> 95% of treatment sites)	Yes	Fully implemented	100%
Kuwait	Yes	No	No	No data available	No data available	Implemented countrywide (> 95% of treatment sites)	Yes	Fully implemented	100%
Oman	Yes	No	No	No data available	Yes, rapid initiation within 7 days of HIV diagnosis	Implemented countrywide (> 95% of treatment sites)	Yes	Implemented countrywide (> 95% of treatment sites)	100%
Qatar	Yes	No	No	No data available	Yes, rapid initiation within 7 days of HIV diagnosis	Implemented countrywide (> 95% of treatment sites)	Yes	Implemented countrywide (> 95% of treatment sites)	No data available
United Arab Emirates (UAE)	Yes	No	No	No data available	No data available	Implemented countrywide (> 95% of treatment sites)	Yes	Partially implemented	No data available

*Bahrain* The country HIV testing strategy includes client-initiated testing and counselling, provider-initiated testing and counselling, routine antenatal testing, community-based testing and counselling, assisted partner notification, and social network-based HIV testing with no self-testing strategy at the present. Currently there are no data reported from a sentinel surveillance system, and no systems are in place to monitor ART resistance. In addition there are no data available about the presence of approved social protection strategy.

*Kuwait* The country has systems in place to monitor antiretroviral drug resistance. Kuwait also has an ongoing social protection strategy. HIV monitoring and evaluation is integrated into a broader health monitoring and evaluation strategy. Sentinel surveillance is conducted in a number of settings; at antenatal clinic, clinics for individuals that inject drugs, prisons and other closed facilities.

*Qatar* Qatar has developed numerous testing strategies including client-initiated testing and counselling, provider-initiated testing and counselling, routine antenatal testing, community-based testing and counselling, assisted partner notification, and social network-based HIV testing. Currently, there is no national policy on HIV self-testing and there is no system in place to monitor ART resistance. There are no data about approved social protection strategy nor are there any data about any ongoing surveillance.

*Oman* The testing strategy includes client-initiated testing and counselling, provider-initiated testing and counselling, routine antenatal testing, and assisted partner notification. Oman uses events such as the Muscat festival, Khareef festival and Awareness campaigns for additional testing and counselling. Oman is implementing social protection strategy/policy/framework. Oman currently has no sentinel surveillance system or system in place to monitor ART drug resistance.

*Saudi Arabia* The HIV testing strategy comprise of client-initiated testing and counselling, provider-initiated testing, and counselling, community-based testing, and counselling, and assisted partner notification. There is a stand-alone HIV monitoring and evaluation strategy. However, the country has no policy on HIV self-testing and does not monitor resistance to ART drugs. The country has ongoing social protection strategy with documented policies and evaluation/framework. Sentinel surveillance is conducted in antenatal clinic, in prisons and other closed door facilities. There are several testing strategies including client-initiated testing and counselling, provider-initiated counselling and testing and, routine antenatal testing, and assisted partner notification. However, there is no policy on HIV self-testing currently.

*United Arab of Emirates (UAE)* The testing strategy includes client-initiated testing and counselling, provider-initiated testing and counselling and routine antenatal testing. However, there are no data available on self-testing and there are no defined systems to monitor ART drug resistance. UAE has an approved social protection strategy/policy/framework which is being implemented. Currently HIV monitoring and evaluation is integrated within a broader health monitoring and evaluation strategy. Sentinel surveillance is conducted at antenatal clinic and at clinics for sex workers.

## Discussion

The GCC countries have a low prevalence of HIV infection and our review documents that the GCC countries have made noticeable progress in achieving some of the UNAIDS targets of 95-95-95 by the year 2025.

With the exception of SA, where the AIDS-related deaths toll in 2021 topped 200, the number of fatalities in the four GCC countries (Oman, Qatar, SA, and UAE) was below 100. The low fatality rate may be related to early case detection, enrolment in ART, and a robust monitoring system.

However, we also found that many GCC countries' were lagging behind in achieving the 95-95-95 targets. While Bahrain, Oman, and UAE appeared to be on track to accomplish the first 95 target by 2025, the situation was less clear for the remaining countries (Kuwait, Qatar and SA).

On the second 95 target i.e. the number of persons who are aware of their HIV status being on ART, we also see modest improvement in Bahrain, Oman, Qatar, and SA. Interestingly to see that there has been a slight increase in the proportion of PLHIV who have received ART and have viral load suppression in two countries (Bahrain and Oman) in the third 95 target. The unsatisfactory rate of viral suppression in GCC countries may be attributable to poor adherence to ART, drug resistance, HIV-related stigma, and family non-disclosure.

However substantive components of the UNAIDS objectives of 95-95-95 by 2025 have been implemented by GCC countries. Firstly, political commitment has been demonstrated through a strong governance and stewardship of the HIV/AIDS Programme within the Ministries of Health with a single administrative structure established to lead the UNAIDS objectives of 95-95-95 strategies. National strategic goals have been established that include access to high-quality HIV services nationwide with the provision of free HIV care services, including ART.

The COVID-19 pandemic has posed a new challenge to the worldwide HIV response, by impacting HIV-related services raising fears that it would reverse the progress made in UNAIDS targets [23, 24]. The COVID-19 pandemic may have enhanced the vulnerability of critical groups to HIV infection and widened the accessibility gap between them and other populations. Disruption of HIV/AIDS surveillance, initiation of ART, and follow-up of individuals on ART during the pandemic, hampered the efficiency of the programs. Thus COVID-19 also had a significant impact on other elements of HIV service adoption, including HIV voluntary counselling and testing, HIV provider-initiated counselling and testing, ART, recently started ART, TB screening, and ART follow-up in Ethiopia [25]. To preserve gains toward HIV epidemic containment, enhancing ART delivery mitigation with the ongoing COVID-19 is recommended as one of the essential health services.

According to the findings of this review, Oman, Qatar and UAE do not fully meet the criteria for the 95-95-95 indicators for all HIV-positive individuals that are aware of their HIV status. This also suggests that a sizeable proportion of PLHIV in these three countries are taking part in the program.

None of the GCC countries met the second and third targets (> 95%); this could be attributed to a sizable portion of PLHIV being unaware of their HIV status and as such not being a part of the program. Furthermore, the COVID 19 pandemic was a significant hindrance to the worldwide 95-95-95 targets [18]. In addition, difficulties with HIV diagnosis testing due to insufficient testing and confidentiality, accessing high risk populations (including non-citizens), and a lack of ART access, as well as treatment non-adherence, may become more prevalent as a result of a variety of factors that endanger both the health of HIV-positive individuals as well as public health, are issues that countries must address.

Surprisingly, according to mathematical models, a 6-month interruption in treatment for half of the PLHIV on ART would result in a 1.63 times (median across models: range 1.39–1.87) increase in HIV-related mortality during a 1-year period compared to no interruptions [26]. To overcome this problem, the national strategic plans have been enabled and access to high-quality HIV services have been maintained with provision of free HIV care services including providing ART. In addition, a national network of laboratory services has been made available. These policies have been adopted and applied across the countries to reduce transmission of infection in healthcare settings. As a step in achieving the 95-95-95 goals, novel techniques to improving access to care need to be implemented. Efforts to improve telemedicine for HIV care should be considered as one of the ways to increase technological support for PLHIV, flexible access options to care, more platforms for remote monitoring of the patients and suitable billing and reimbursement mechanisms [27]. Furthermore, clinical informatics and machine learning models have the potential to improve HIV care continuum outcomes. Clinical decision support systems and electronic warnings based on electronic medical records have been used effectively to improve HIV care continuum outcomes [28].

### Moving Forward to Achieve Global Targets

The first and foremost step for the HIV/AIDS program should be to regularly assess difficulties and progress with a focus on the major issues that could hinder reaching the 95-95-95 targets by 2025. Some of the key actions include: (1) the urgent requirement for the establishment of an appropriate national task force for assessment of the commitment to achieve the 95-95-95 targets by 2030 and effective systematic monitoring; (2) As the most important objective is still the first objective, in which the health center as well as including private sectors institutions should identify all possible HIV-positive individuals (citizen and non-citizen), with a focus on key populations, have them tested for HIV, and immediately start HIV-positive patients on antiretroviral therapy (ART) while monitoring treatment through viral load testing and patient follow-ups; (3) the countries need to bolster initiatives including community awareness, voluntary counselling, testing campaigns; (4) health care workers (HCWs) need to be trained and made more aware of how to actively find HIV cases, especially in high-risk groups; (5) to reach the UNAIDS 95-95-95 SDG targets, health facilities and institutions must follow the WHO and UNAIDS standards for HIV treatment (test and treat); (6) ongoing and appropriate HIV/AIDS management and strengthening of HIV prevention, care, and control services, together with systematic HIV testing and treatment, should be addressed for non-nationals; (7) it is necessary to design a functional monitoring scheme with specified 5-year national and governorate operational goals and milestones; (8) engagement of private sector institutions and a focus on vulnerable populations, such as noncitizen populations, were emphasized as ongoing issues that need resolution through the doable and practical interventions strategies; (9) at the governorate (provincial) level, an effective supervision and monitoring system needs to be set up; (8) reports on accomplishments and lessons learned must be shared with policymakers, healthcare workers, and other stakeholders; (10) service integration and decentralization.

### Review Study Limitations

Our review has a number of limitations. First, due to the lack of data it was difficult to estimate the overall number of PLHIV who know their HIV status. Also we only analysed secondary data from patient records reported by the countries and thus could only analyse and interpret data/variables using those characteristics that are regularly reported by the countries. No information on the proportion of PLHIV who initially knew their status by gender, age, and geographic area were available for collation. Missing information mandates that data are viewed with caution. In addition, we were able to disaggregate the data into citizens and non-citizens.

## Conclusions

The GCC countries have made significant strides toward achieving the 95-95-95 targets, but there is still a long way to go to accomplish the UNAIDS targets. The review proposes critical options for enhancing delayed HIV care presentation. The GCC countries must strive diligently to accomplish the 95-95-95 targets by emphasising early identification of the cases by increasing testing and screening as well as initiation of ART therapy and monitoring viral load suppression. In addition, achieving the 95-95-95 targets will not be achievable until the response is targeted to the needs of key populations while addressing non-citizens and overcoming operational hurdles in order to implement the program as successfully as possible.

## Data Availability

All raw data are available upon reasonable request.
