# Correction: Progress of the Gulf Cooperation Council (GCC) Countries Towards Achieving the 95–95–95 UNAIDS Targets: A Review

**DOI:** 10.1007/s44197-023-00129-w

**Published:** 2023-06-22

**Authors:** Salah Al Awaidy, Ramy Mohamed Ghazy, Ozayr Mahomed

**Affiliations:** 1 Office of Health Affairs, Ministry of Health, Muscat, Oman; 2 Tropical Health Department, High Institute of Public Health, Alexandria University, Alexandria, Egypt; 3 Department of Public Health Medicine, University of KwaZulu Natal, Durban Howard College Campus, South Africa; 4 Dasman Diabetes Institute, Kuwait City, Kuwait

**Correction to: Journal of Epidemiology and Global Health (2023)** 10.1007/s44197-023-00097-1

In the original version of this article, the middle name and ORCiD of Ramy Mohamed Ghazy were missing and the sentence ‘The figures were built using Microsoft Office 2016 and ggplot2 package built under R version 4.2.’ should have read ‘The figures were built using Microsoft Office 2016.’

The original article has been corrected.

